# Early Clinical, Laboratory, and Imaging Correlates of Neurological Dysfunction in Adults Presenting to the Emergency Department with Sepsis: A Single-Center Retrospective Study

**DOI:** 10.14789/ejmj.JMJ26-0022-OA

**Published:** 2026-04-10

**Authors:** FLORIN SCARLATESCU, ECATERINA SCARLATESCU, DANIELA BARTOS

**Affiliations:** 1 Department of Neurology, Clinical Emergency Hospital, Bucharest, Romania; 1 Department of Neurology, Clinical Emergency Hospital, Bucharest, Romania; 2 University of Medicine and Pharmacy Carol Davila, Bucharest, Romania; 2 University of Medicine and Pharmacy Carol Davila, Bucharest, Romania; 3 Department of Anaesthesia and Intensive Care, Fundeni Clinical Institute, Bucharest, Romania; 3 Department of Anaesthesia and Intensive Care, Fundeni Clinical Institute, Bucharest, Romania

**Keywords:** sepsis-associated encephalopathy, neurological dysfunction, red cell distribution width, brain CT, cortical atrophy

## Abstract

**Objectives:**

To characterize the prevalence and clinical profile of neurological dysfunction and pure sepsis-associated encephalopathy (SAE) in adults presenting to the emergency department (ED) with sepsis, and to identify early clinical, laboratory, and brain imaging parameters associated with these phenotypes.

**Materials:**

This retrospective study included 226 adults with sepsis (Sepsis-3 criteria) at a tertiary emergency hospital. Neurological dysfunction was defined as an acute mental status change, and pure SAE was diagnosed after systematic exclusion of other identifiable causes of encephalopathy. Demographic, clinical, laboratory, and brain computed tomography (CT) data were collected from medical records.

**Methods:**

Group comparisons were made using Mann-Whitney U, chi-square, or Fisher’s exact tests. Multivariable binary logistic regression identified predictors of neurological dysfunction and pure SAE.

**Results:**

Neurological dysfunction was present in 79.6% of patients, and pure SAE in 24.8%. In the multivariate model, red cell distribution width (RDW) independently predicted pure SAE (OR 1.20, 95% CI 1.05-1.36; p = 0.005), while creatinine was inversely associated (OR 0.77, 95% CI 0.60-0.99; p = 0.038), consistent with the exclusion of patients with metabolic causes of encephalopathy from the pure SAE category. Frontal cortical atrophy on brain CT was the only imaging parameter linked to pure SAE (p = 0.022). Neurological dysfunction and pure SAE were associated with lower survival at discharge (26% vs. 67.3% and 19.6% vs. 39.4%, respectively).

**Conclusions:**

Neurological dysfunction is common in ED sepsis patients. RDW and frontal cortical atrophy may help identify pure SAE early. These markers could aid rapid risk stratification and management.

## Introduction

Sepsis-associated encephalopathy (SAE) refers to widespread neurological dysfunction resulting from an abnormal systemic response to severe infections or sepsis, occurring in the absence of direct central nervous system infection, acute brain injury, or alternative causes of encephalopathy^1)^. SAE presents with a broad spectrum of neurological symptoms, from mild confusion, hypersomnolence, and delirium to more severe states such as stupor or coma. This heterogeneity, coupled with the lack of specific biomarkers, contributes to frequent underrecognition and diagnostic uncertainty in routine clinical practice^2)^.

Sepsis-associated encephalopathy (SAE) can be classified into two types: an early-onset form, which often precedes other organ dysfunction or overt signs of infection, and a later-onset form. The later type is multifactorial, resulting not only from sepsis itself, but also from factors such as multiple organ failure, sedative medications, and the conditions associated with intensive care^3)^. Diagnosing early-onset SAE is particularly challenging, because neurological dysfunction may be the sole or leading manifestation at the time of evaluation in the emergency department (ED), and the clinical picture—usually an acute alteration in mental status without focal deficits or meningeal signs—is highly nonspecific. In this setting, SAE must be distinguished from a wide array of alternative diagnoses, including metabolic, toxic, structural, and primary neurological disorders, and a high index of suspicion is required to ensure timely recognition and appropriate management^2)^.

The reported prevalence of brain dysfunction among patients with sepsis reaches up to 70%, although estimates vary considerably across studies due to differences in inclusion criteria^4, 5)^. In a recent investigation, sepsis-associated encephalopathy (SAE) was identified in 411 out of 603 patients admitted to the intensive care unit (ICU) for sepsis secondary to pulmonary infection^6)^. The majority of available literature pertains to ICU populations, while studies addressing brain dysfunction in patients at the point of hospital admission—regardless of illness severity or subsequent ICU transfer—remain limited.

At present, there are no universally accepted diagnostic criteria for SAE, and diagnosis remains largely one of exclusion. In practice, SAE is established after ruling out alternative causes of encephalopathy through clinical evaluation, basic laboratory testing, and, where appropriate, neuroimaging and cerebrospinal fluid analysis. In many patients, a thorough neurological examination combined with routine laboratory investigations is sufficient to support the diagnosis. Neuroimaging is generally reserved for a minority of patients, primarily to exclude cerebrovascular events or traumatic brain injury. For instance, a pediatric study reported that 21% of children with sepsis exhibited brain lesions detectable by magnetic resonance imaging (MRI)^7)^. In adult ICU populations with traumatic brain injury, MRI has revealed both new cerebral lesions and the extension of existing injuries in the context of infection and sepsis^8)^. While MRI is commonly utilized in research on sepsis, there remains a paucity of data concerning the diagnostic and prognostic value of brain computed tomography (CT) findings at the time of hospital admission, particularly during the very early phase of sepsis.

Despite growing interest in SAE, several important gaps remain. First, most studies focus on ICU patients and later timepoints, when neurological status is confounded by sedation, prolonged organ support, and cumulative iatrogenic factors. Second, few investigations distinguish “pure” SAE—defined as encephalopathy primarily attributable to sepsis—from multifactorial encephalopathy driven by concurrent renal, hepatic, or other metabolic derangements. Third, there is limited evidence on simple, bedsideavailable clinical, laboratory, and imaging markers that can help clinicians differentiate and predict SAE early, at the time of ED presentation, when diagnostic and therapeutic decisions are most consequential.

Against this background, the present study was designed with two main objectives. First, to characterize the prevalence and clinical profile of neurological dysfunction and pure SAE in adults presenting to the ED with sepsis, independently of subse quent ICU admission. Second, to identify early clinical, laboratory, and brain imaging parameters—readily available at or shortly after ED arrival—that are associated with neurological dysfunction at admission and with pure SAE, and to develop multivariable models capable of predicting these phenotypes early in the hospital course. By focusing on the ED setting and explicitly separating pure SAE from multifactorial encephalopathy, this research addresses a critical gap in the current literature and aims to provide clinicians with practical tools to recognize SAE promptly, refine etiological attribution of altered mental status, and potentially guide risk stratification and early management in routine clinical practice.

## Materials and Methods

### Patient selection

This retrospective observational study enrolled patients with sepsis, as defined by the Sepsis-3 criteria^9)^, following confirmation of diagnosis in the emergency department. Data were collected from Clinical Emergency Hospital Bucharest, a high-volume tertiary care center in Romania. The study protocol received ethical approval from the institutional ethics committee (approval number 2257/22. 01.2024), which waived informed consent requirements owing to the retrospective, non-interventional design and reliance on routinely collected clinical data. In accordance with institutional policy, all patients provide written consent for the use of clinical data obtained during their hospital admission.

### The diagnosis of neurological dysfunction at admission and pure SAE

Neurological dysfunction at admission was defined as an acute alteration in mental status—including confusion, disorientation, a reduced level of consciousness, agitation or delirium—detected during the initial clinical evaluation in the ED. The diagnoses of neurological dysfunction at admission and pure SAE were established by the neurologist examining the patients. A diagnosis of pure SAE was established after systematic exclusion of other identifiable causes of neurological impairment, including direct central nervous system infection (meningitis, encephalitis, brain abscess), acute cerebrovascular events, traumatic brain injury, severe hepatic encephalopathy in patients with cirrhosis, severe uremic encephalopathy in patients with advanced renal failure, primary metabolic derangements (e.g. hypoglycemia, severe electrolyte disturbances) judged to be the dominant cause, drug-related causes (sedation, intoxication), and pre-existing neuropsychiatric conditions that could fully account for the clinical picture. In patients where encephalopathy was considered multifactorial—i.e. driven by a combination of sepsis and concurrent organ failures such as uremia or hepatic insufficiency—the episode was not classified as pure SAE.

Demographic and clinical data were retrieved from the electronic medical records for all enrolled patients and included age, sex, pre-existing comorbidities (arterial hypertension, atrial fibrillation, heart failure, diabetes mellitus, chronic kidney disease, cirrhosis, active cancer and metastatic cancer), the source and microbiological profile of infection, length of stay in the intensive care unit (ICU) and in the hospital, and survival at hospital discharge.

### Blood tests

The blood samples were obtained from all patients in the ED at the time of initial presentation, before initiation of fluid resuscitation or antibiotic therapy in most cases. The following parameters were recorded: white blood cell count (WBC), hemoglobin (Hb), mean corpuscular volume (MCV), platelet count (PLT), platelet distribution width (PDW), red cell distribution width (RDW), mean platelet volume (MPV), glucose, serum creatinine, blood urea, serum sodium, and international normalized ratio (INR). All analyses were performed using the hospital’s central laboratory, with automated hematology and biochemistry analyzers calibrated according to standard institutional protocols.

### Imaging

Brain computed tomography (CT) was performed during the initial hospital admission in a subset of patients, according to clinical indication (e.g. acute alteration of consciousness, focal neurological signs, or clinical suspicion of intracranial pathology). CT imaging was the method used to rule out acute intracerebral lesions, but in the same time the CT findings were explored to identify simple imaging markers associated with SAE.

All available CT scans were retrospectively reviewed by a single investigator (neurologist, SF), and the following structural brain parameters were systematically recorded: total number of cerebral lacunes and macroinfarcts; third ventricle diameter (measured in millimeters at the widest point on axial images); cortical atrophy: graded separately for each lobe (frontal, temporal, parietal, occipital) and as a global cortical atrophy (GCA) score using a 4-point visual rating scale (0 = no atrophy, 1 = mild, 2 = moderate, 3 = severe)^10)^; white matter disease: graded using the Fazekas scale (0 = absent, 1 = punctate foci, 2 = beginning confluent, 3 = confluent periventricular white matter hyperintensities)^11, 12)^; medial temporal atrophy (MTA): scored according to the Scheltens visual rating scale (0-4), where 0 indicates no atrophy and 4 indicates severe medial temporal lobe atrophy with marked widening of the choroid fissure and temporal horn^13, 14)^; ventricular indices as markers of brain atrophy: the bicaudate index (ratio of the minimum intercaudate distance to the brain width at the same level) and the Evans index (ratio of the maximum width of the frontal horns to the maximum internal diameter of the skull) were calculated on axial images as quantitative markers of central brain atrophy and ventricular enlargement^15-17)^.

### Statistical analysis

Continuous variables were tested for normality using the Shapiro-Wilk test. Data with normal distribution were reported as mean ± SD. As the majority of laboratory and imaging parameters showed non-normal distributions, continuous and ordinal data were expressed as median (interquartile range) and compared between groups using the Mann-Whitney U test. Categorical and binary variables were expressed as absolute frequencies and percentages and compared using the chi-square test or Fisher’s exact test, as appropriate. For the identification of independent predictors of neurological dysfunction at admission and pure sepsis- associated encephalopathy, variables with a p-value below 0.10 in the univariate analysis were entered into multivariable binary logistic regression models. Results of the logistic regression are reported as adjusted odds ratios (OR) with 95% confidence intervals (CI). Multicollinearity among predictors entered in the logistic regression model was assessed in SPSS by running a separate linear regression with these variables entered as independent predictors and requesting collinearity diagnostics (tolerance, variance inflation factor, and collinearity diagnostics table). For this study, a p value of < 0.05 was considered significant. Statistical analyses were performed using SPSS Statistics v 23.0 (IBM Corp).

## Results

A total of 226 patients (42.9% men, 57.1% women) with sepsis were included in the analysis. The mean (± SD) age was 72.69 (± 15.07) years. Overall neurological dysfunction at admission was very common, affecting nearly 80% of patients (n = 180). Pure SAE was identified in approximately one quarter of patients (n = 56, 24.8%).

### Baseline characteristics

Patients with and without neurological dysfunction at admission had similar age (mean age 72.54 vs 73.26 years). Patients with pure SAE were slightly younger than those without (mean age ≈70.5 vs 73.3 years), although the difference was modest. Sex distribution was similar between groups, with men representing 42.2% and 44.6% of the cases with neurological dysfunction at admission and with pure SAE, respectively. Patients with and without neurological dysfunction at admission had no statistically significant differences in hypertension, atrial fibrillation, heart failure, diabetes, cirrhosis, cancer, or metastatic cancer incidence. Chronic kidney disease tended to be more frequent in patients with neurological dysfunction at admission, but this difference also did not reach conventional significance (Table 1).

When patients with and without pure SAE were compared, age and most comorbidities again showed no significant variation between groups. However, chronic kidney disease was significantly less common in patients with pure SAE, whereas cirrhosis was observed only in patients without this diagnosis, consistent with the clinical attribution of their associated neurological dysfunction to hepatic or multifactorial mechanisms (Table 1).

**Table 1:** Baseline demographic and comorbidity characteristics of sepsis patients stratified by neurological dysfunction at admission and pure sepsis-associated encephalopathy

	Neurological dysfunction at admission		P value	Pure sepsis-associated encephalopathy		P value
	YES (n = 180)	NO (n = 46)		YES (n = 56)	NO (n = 170)	
Age (years)	72.5(± 15.3)	73.2 (± 14.9)	0.771	70.5 (± 16.7)	73.3 (± 14.4)	0.240
AHT	92 (82.1%)	20 (43.4%)	0.350	26 (46.4%)	86 (50.5%)	0.589
AF	70 (38.9%)	12 (26.1%)	0.10	16 (28.5%)	66 (38.8)	0.166
HF	49 (27.2%)	13 (28.3%)	0.880	14 (25%)	48 (28.2%)	0.638
CKD	58 (32.2%)	9 (19.5%)	0.091	8 (14.2%)	59 (34.7%)	0.004*
DM	55 (30.5%)	18 (39.1%)	0.262	16 (28.5%)	57 (33.5%)	0.490
Cirrhosis	8 (4.4%)	2 (4.3%)	0.977	0 (0%)	10 (5.9%)	0.016*
Cancer	31 (17.2%)	11 (23.9%)	0.298	14 (25%)	28 (16.4%)	0.155
Metastatic cancer	10 (5.8%)	5 (10.9%)	0.196	5 (8.9%)	10 (5.9%)	0.427

Data are expressed as mean ± SD or n (%). P value was obtained using the t-test or chi-square test, as appropriate. * p statistically significant (< 0.05); AHT, arterial hypertension; AF, atrial fibrillation; HF, heart failure; CKD, chronic kidney disease; DM, diabetes mellitus.

### Organ dysfunction, laboratory data and outcomes

Among patients with neurological dysfunction at admission, hemoglobin, leukocyte and platelet counts were comparable to those without neurological involvement, but red cell distribution width (RDW) and mean platelet volume (MPV) were higher, suggesting differences in hematologic activation. The patients presenting neurological dysfunction at admission also exhibited more pronounced renal dysfunction, with higher creatinine and urea, as well as higher sodium and INR, consistent with broader organ failure. Neurological dysfunction at admission was associated with a longer intensive care stay, while overall hospital length of stay did not differ significantly (Table 2).

When stratified by pure SAE, standard hematologic parameters and platelet indices showed no major differences between groups. In contrast, patients with SAE had better preserved renal function, with lower creatinine and urea values, and a shorter hospital stay compared with those without this diagnosis, whereas ICU length of stay was similar (Table 2).

**Table 2:** Laboratory parameters, and clinical outcomes of sepsis patients stratified by neurological dysfunction at admission and pure sepsis-associated encephalopathy

Parameter	Reference ranges	Neurological dysfunction at admission		P value	Pure sepsis-associated encephalopathy		P value
		YES (n = 180)	NO (n = 46)		YES (n = 56)	NO (n = 170)	
WBC (/µL)	4000-10000	15000 (12430)	15650 (14980)	0.825	13700 (14125)	15700 (12585)	0.648
Hemoglobin (g/dL)	11.7-16	11.3 (4)	12.2 (3)	0.130	11.4 (4)	11.5 (4)	0.339
RDW (%)	11.5-14.5	14.4 (3)	13.3 (2)	0.026*	14.6 (3)	14.2 (3)	0.099
Platelet count (*1000/µL)	150-400	193.5 (140)	222.5 (131)	0.200	214 (118)	194.5 (144)	0.801
PDW (%)	9-17	17.6 (2)	17.3 (2)	0.137	17.4 (2)	17.6 (2)	0.448
MPV (fL)	7-11	8.4 (3)	7.4 (4)	0.034*	7.9 (3)	8.4 (3)	0.887
Creatinine (mg/dL)	0.55-1.02	2 (2)	1.3 (2)	0.001*	1.24 (1)	2.25 (2)	<0.001*
Urea (mg/dL)	19-49	103 (112)	69.5 (44)	0.001*	58.5 (52)	103 (111)	<0.001*
Sodium (mmol/L)	135-145	139.9 (12)	138 (10)	0.03*	139 (11)	139 (11)	0.569
INR	0.9-1.2	1.38 (1)	1.21 (0)	0.008*	1.38 (0)	1.31 (1)	0.371
ICU LOS (days)		2 (6)	0 (6)	0.031*	2 (6)	1.5 (6)	0.326
Hospital LOS (days)		5 (12)	8 (8)	0.23	3.5 (8)	7 (12)	0.023*
Survival at hospital discharge		47 (26%)	31 (67.3%)	<0.001*	11 (19.6%)	67 (39.4%)	<0.007*

Data are expressed as median (IQR) or n (%). P value was obtained using the Mann Whitney U test or chi-square test, as appropriate. * p statistically significant (< 0.05); WBC, white blood cells; RDW, red blood cells distribution width; PDW, platelet distribution width; LOS, length of stay

In the multivariate model restricted to laboratory and hematologic parameters, none of the candidate variables (RDW, MPV, creatinine, urea, sodium, INR) showed an independent association with neurological dysfunction at admission at the conventional significance threshold. The point estimates suggested only modest effects, and confidence intervals were wide and crossed unity, indicating that, once their inter correlations were taken into account, these indices did not reliably distinguish patients with and without neurological dysfunction at hospital admission (Table 3).

**Table 3:** Multivariable logistic regression analysis of independent predictors of neurological dysfunction at admission and pure sepsis-associated encephalopathy

Predictor	Neurological dysfunction at admission OR (95% CI)	P value	Sepsis-associated encephalopathy OR (95% CI)	P value
RDW (%)	1.16 (0.96-1.39)	0.124	1.20 (1.05-1.36)	0.005*
MPV (fL)	1.11 (0.93-1.34)	0.255		
Creatinine (mg/dL)	1.11 (0.81-1.52)	0.506	0.77 (0.60-0.99)	0.038*
Urea (mg/dL)	1.01 (1.00-1.02)	0.06	1.00 (0.99-1.00)	0.123
Sodium (mmol/L)	1.03 (0.99-1.08)	0.139		
INR	1.13 (0.74-1.72)	0.574		

RDW, red blood cells distribution width; MPV, mean platelet volume; * p statistically significant (<0.05)

In contrast, the model for pure SAE identified RDW and creatinine as independent predictors. For RDW, the adjusted odds ratio of approximately 1.20 per unit increase (95% CI roughly 1.05-1.36) indicates that for each 1% increment in RDW the odds of sepsis associated encephalopathy increased by about 20%, after controlling for the other laboratory variables in the model. Creatinine showed an opposite pattern, with an adjusted odds ratio of about 0.77 per 1 mg/dL (95% CI around 0.60-0.99), meaning that each 1 mg/dL increase in creatinine was associated with a 23% reduction in the odds of being classified as having pure SAE. This inverse association supports the interpretation that patients with more severe renal impairment are less often categorized as having pure SAE and are instead considered to have multifactorial or metabolically driven encephalopathy. Urea did not remain independently significant once RDW and creatinine were included in the model. Of note, creatinine and urea showed moderate collinearity (Spearman rho = 0.688), with variance inflation factors of approximately 2 for both variables, which may have contributed to the attenuation of the urea effect in the adjusted model while not invalidating the regression estimates (Table 3).

### Brain imaging findings

CT scan was performed in 92 patients, and the imagistic findings were compared between patients with and without neurological dysfunction at admission and pure SAE. Among the 92 patients with available brain CT, 84 had neurological dysfunction at admission and 8 did not; 26 were classified as having pure SAE and 66 were not.

The number of cerebral lacunes, macroinfarcts, and third ventricle diameter were similar between patients with and without neurological dysfunction at admission, with no statistically significant differences. Likewise, the bicaudate index and Evans index did not differ between groups. Among the cortical atrophy scores, temporal cortical atrophy showed a trend towards more severe grades in patients with neurological dysfunction, approaching conventional significance, while global cortical atrophy showed a weaker, non-significant trend in the same direction. No significant differences were found for frontal, parietal, or occipital cortical atrophy, nor for medial temporal atrophy (MTA Scheltens) or Fazekas scale scores (Table 4).

When patients were stratified by pure SAE, the number of lacunes, macroinfarcts, third ventricle diameter, bicaudate index, and Evans index again showed no significant differences between groups. Among the regional cortical atrophy scores, only frontal cortical atrophy demonstrated a significantly different distribution between patients with and without pure SAE. Notably, this difference was driven by a markedly lower proportion of grade 1 (mild) frontal atrophy in patients with SAE compared with those without, alongside a shift towards higher grades of atrophy (grades 2 and 3), suggesting a bimodal distribution in the SAE group rather than a simple linear increase in atrophy severity. Global, temporal, parietal, and occipital cortical atrophy, as well as medial temporal atrophy and Fazekas scale scores, did not differ significantly between the two groups (Table 4)

Taken together, these imaging findings indicate that the burden of chronic cerebrovascular and neurodegenerative lesions detectable on routine brain CT was broadly similar between patients with and without neurological dysfunction at admission or pure SAE. The only significant imaging association identified was between frontal cortical atrophy and pure SAE, suggesting that pre-existing frontal lobe vulnerability may contribute to the clinical expression of this phenotype, although this finding should be interpreted cautiously given the relatively small imaging subgroup and the multiple comparisons performed.

**Table 4:** Brain CT imaging findings in sepsis patients stratified by neurological dysfunction at admission and pure sepsisassociated encephalopathy

Parameter		Neurological dysfunction at admission		P value	Pure sepsis-associated encephalopathy		P value
		YES (n = 84)	NO (n = 8)		YES (n = 26)	NO (n = 66)	
Number of cerebral lacunes		1(3)	0.5 (6)	0.834	1 (3)	1 (3)	0.724
Number of macroinfarcts		0 (1)	0 (0)	0.318	0 (1)	0 (1)	0.854
V3 diameter (mm)		11.3 (4.9)	10.5 (5.3)	0.708	10.5 (7)	11.5 (4.3)	0.706
Bicaudat index		0.2 (0.06)	0.19 (0.1)	0.978	0.19 (0.09)	0.2 (0.06)	0.673
Evans index		0.3 (0.07)	0.32 (0.07)	0.311	0.3 (0.07)	0.3 (0.08)	1
Global cortical atrophy	0	13	2	0.128	6	9	0.589
	1	17	4		4	17	
	2	44	2		13	33	
	3	10	0		3	7	
Frontal cortical atrophy (Frontal GCA)	0	15	2	0.279	7	10	0.022*
	1	22	4		2	24	
	2	38	2		13	27	
	3	9	0		4	5	
Temporal cortical atrophy (Temporal GCA)	0	15	2	0.093	6	11	0.622
	1	23	5		6	22	
	2	39	1		11	29	
	3	7	0		3	4	
Parietal cortical atrophy (Parietal GCA)	0	16	3	0.488	6	13	0.443
	1	26	3		7	22	
	2	38	2		13	27	
	3	3	0		0	3	
Occipital cortical atrophy (Occipital GCA)	0	36	5	0.567	10	31	0.694
	1	32	2		10	24	
	2	16	1		6	11	
	3	0	0		0	0	
MTA Scheltens	0	13	2	0.385	6	9	0.755
	1	21	4		5	20	
	2	30	1		9	22	
	3	16	1		5	12	
	4	4	0		1	3	
Fazekas Scale	0	22	2	0.988	8	16	0.714
	1	33	3		9	27	
	2	16	2		4	15	
	3	12	1		5	8	

Data are expressed as median (IQR) or n (number of patients). p value was obtained using the Mann Whitney U test or chi-square test, as appropriate. * p statistically significant (< 0.05)

## Discussion

The present study found that nearly 80% of adults presenting to the ED with sepsis had neurological dysfunction at admission, a prevalence substantially higher than the commonly cited range of 8-70% from earlier ICU-based series^4)^. This difference is likely explained by the inclusive definition adopted in this study, which captured the full spectrum of altered mental status—from mild confusion and disorientation to coma—at the very first clinical contact, before any therapeutic intervention. Previous estimates were typically generated in ICU cohorts, at later time-points when sedation, fluid resuscitation, or metabolic correction may have already attenuated or confounded neurological signs. When a strict exclusion-based definition was applied to identify pure SAE—attributing encephalopathy primarily to sepsis after ruling out hepatic, uremic, metabolic, structural, and drug-related causes—the prevalence fell to 24.8%. This figure is consistent with the lower end of reported SAE incidence (15.0-62.4%) in the recent systematic review by Zhou et al. (2025)^18)^, which documented wide variability across studies depending on diagnostic criteria, patient populations, and care settings. Notably, the strict exclusion of cirrhotic patients (none classified as pure SAE), patients with severe renal impairment, and those with multifactorial encephalopathy likely accounts for the relatively conservative prevalence of pure SAE in the present cohort.

An important finding of this study is the substantially worse survival in patients with neurological dysfunction at admission (26% vs. 67.3%) and in those with pure SAE (19.6% vs. 39.4%). These figures are in line with the seminal report by Sprung et al. (1990), which first demonstrated that the mortality rate of septic patients with altered mental status was 49% compared with 26% in those with normal mental status^19)^. In a large international post hoc analysis of the ICON audit encompassing 7,192 critically ill patients, Crippa et al. confirmed that brain failure combined with sepsis was independently associated with a markedly increased risk of hospital death (adjusted RR 5.61, 95% CI 3.93-8.00), substantially higher than either sepsis or brain failure alone^20)^. Sonneville et al. (2017), in a retrospective analysis of 2,513 patients, found that even mild alterations of mental status (GCS 13-14) at ICU admission were independently associated with increased mortality (HR 1.38, 95% CI 1.09-1.38), while severe encephalopathy (GCS 3-8) carried a hazard ratio of 3.37 (95% CI 2.82-4.03)^21)^. A 2026 systematic review of risk prediction models for SAE confirmed that 28-day mortality in SAE can reach up to 46%, and that SAE prevalence ranges from 8% to 70% depending on diagnostic criteria^22)^. Shen et al. (2025) extended these findings by demonstrating that dynamic GCS trajectories over the first 5 ICU days carry distinct prognostic implications—patients with persistently high GCS scores had better survival compared to those with decreasing GCS scores^23)^. The current study extends this body of evidence by demonstrating that neurological dysfunction, assessed at the earliest clinical contact in the ED—before sedation, fluid resuscitation, or ICU-related confounders—already carries strong prognostic significance regardless of subsequent ICU admission. This early-assessment perspective is particularly important because the vast majority of prior studies examined prognosis using neurological evaluations performed after ICU admission, when the clinical picture is frequently confounded by iatrogenic factors.

Among all laboratory parameters evaluated, RDW emerged as the only independent positive predictor of pure SAE in the multivariate model, with each 1% increment in RDW being associated with a 20% increase in the odds of having pure SAE after controlling for creatinine and urea. This finding adds to a growing body of evidence linking elevated RDW to sepsis severity and organ dysfunction. A meta-analysis by Zhang et al. (2020), encompassing 17,961 sepsis patients across 11 studies, demonstrated that increased baseline RDW was significantly associated with mortality (pooled HR 1.14, 95% CI 1.09-1.20)^24)^. Similarly, Singh et al. demonstrated that RDW had excellent diagnostic accuracy (AUC = 0.823) for predicting sepsis mortality, performing comparably to serum lactate^25)^. The biological plausibility of the RDW-SAE association is well-established. Sepsis induces a state of systemic inflammation with markedly increased oxidative stress, which profoundly affects erythrocyte biology. Beyond reflecting systemic inflammation, elevated RDW may also indicate impaired erythrocyte deformability and microcirculatory dysfunction, which are increasingly recognized as contributors to organ injury in sepsis. Inflammatory cytokines and oxidative stress can disrupt erythrocyte membrane integrity and cytoskeletal stability, resulting in heterogeneous red blood cell populations and reduced deformability^26)^. These alterations may impair capillary transit and oxygen delivery in the microcirculation^27)^. Because cerebral perfusion relies on efficient microvascular flow, disturbances in red blood cell rheology could contribute to regional cerebral hypoxia and metabolic stress in vulnerable brain regions. Microcirculatory alterations, including endothelial activation and heterogeneous capillary perfusion, are well-recognized features of sepsis and have been implicated in the pathogenesis of SAE^28)^. In this context, RDW may represent a readily available surrogate marker of systemic processes affecting cerebral microcirculatory integrity^24)^. However, RDW has also been associated with overall disease severity in critically ill patients. Therefore, the observed association with SAE may partly reflect a greater systemic inflammatory burden, and future studies incorporating established severity indicators such as SOFA score or lactate levels will be necessary to clarify its independent predictive value.

Pro-inflammatory cytokines—including TNF-α, IFN-γ, IL-1β, and IL-6—suppress erythroid progenitor cell activity, impair erythrocyte deformability, and alter RBC membrane structure, resulting in anisocytosis and measurably increased RDW^26)^. Recent in-vitro and in-silico evidence has confirmed that oxidative stress is a direct mechanistic driver of increased RDW^29)^. It is therefore conceivable that the heightened systemic inflammation reflected by elevated RDW also drives the neuroinflammatory cascades underlying SAE, including blood-brain barrier disruption, microglial activation, and neuronal dysfunction^30)^. From a clinical perspective, RDW is universally available, inexpensive, and reported as part of a standard complete blood count. Its identification as an independent predictor of pure SAE in this study supports its potential utility as an early bedside marker for SAE risk stratification in the ED, where more sophisticated biomarkers (S100β, NSE) or neuroimaging are often not immediately available. A recent systematic review of SAE prediction models noted that while age, SOFA score, and serum sodium were the most frequently included predictors, RDW has not yet been incorporated into published SAE prediction tools, representing a novel and readily implementable addition^18)^.

Creatinine showed an inverse association with pure SAE in the multivariate model (OR 0.77, 95% CI 0.60-0.99; p = 0.038), a finding that is counterintuitive at first glance but is fully consistent with the study’s diagnostic framework. Patients with more severe renal impairment—manifested by higher creatinine and urea levels—were systematically excluded from the pure SAE category if their encephalopathy was judged to be predominantly uremic or multifactorial in origin. Thus, the lower creatinine observed in the pure SAE group does not imply that preserved renal function causes SAE; rather, it reflects the clinical attribution process whereby encephalopathy in patients with advanced renal dysfunction is classified as multifactorial rather than as pure sepsis-driven. This interpretation is supported by the univariate findings: creatinine and urea were both significantly higher in patients with neurological dysfunction at admission overall (p = 0.001 for both), consistent with the well-recognized role of acute kidney injury as a contributor to neurological impairment in sepsis. Sepsis-associated acute kidney injury is itself a highly prevalent complication, and the interplay between renal failure and cerebral dysfunction is complex, involving accumulation of uremic toxins, altered drug clearance, and compounding metabolic derangements^31)^. The present findings underscore the importance of carefully distinguishing the etiological attribution of encephalopathy in clinical practice, as the management implications of pure SAE versus uremic or hepatic encephalopathy may differ substantially.

Regarding the imagistic findings, among the 92 patients who underwent brain CT, frontal cortical atrophy was the only imaging parameter significantly associated with pure SAE (p = 0.022). This resonates with accumulating evidence that pre-existing brain vulnerability—whether from neurodegeneration, cerebrovascular disease, or atrophy—predisposes to delirium and acute brain dysfunction during systemic illness. Pendlebury et al. demonstrated that white matter changes and cerebral atrophy on brain imaging obtained up to 5 years earlier predicted delirium during subsequent hospital admissions in a stroke/TIA cohort^32)^. The frontal lobes are particularly relevant given their role in attention, executive function, and behavioral regulation—the very cognitive domains most affected in SAE and delirium. Functional MRI studies in sepsis survivors have specifically identified decreased degree centrality and nodal efficiency in frontal regions (particularly the right orbital inferior frontal gyrus), suggesting that the frontal lobe is a key node of vulnerability in sepsis-associated neurological injury^33)^. Nakae et al. reported rapidly progressive brain atrophy in septic ICU patients using semiautomatic CT volumetry, with a mean brain volume decrease of 3.7% over a median of 31 days^34)^. While the present study assessed pre-existing atrophy on a single CT obtained at admission rather than progressive volume loss, the two findings are complementary: patients who already have frontal atrophy may be more susceptible to SAE at presentation, and sepsis may subsequently accelerate further brain volume loss.

The present study’s approach—focusing on simple, routinely available ED parameters including RDW and brain CT findings—complements existing SAE prediction models, which have predominantly been developed in ICU settings using large administrative databases^18)^. A notable distinction of the present study is its focus on the ED setting, which shifts the clinical perspective from predicting SAE among established ICU patients to identifying neurological dysfunction at the point of first medical contact. This is clinically meaningful because the ED represents the earliest window of opportunity for recognition and intervention, before sedation, mechanical ventilation, and other ICU-related confounders obscure the neurological picture. Additionally, the explicit separation of pure SAE from multifactorial encephalopathy represents a conceptual refinement that few prior studies have attempted, yet one that has clear clinical implications for diagnostic work- up and management^35)^. Recently, Tian et al. developed a predictive model for SAE based on immunological markers (IFN-γ, TNF-α, and CD4+/CD8+ ratio), demonstrating the potential of immune biomarkers in SAE prediction^36)^. While such markers may offer superior pathophysiological specificity, they are not routinely available in most EDs. The identification of RDW—a universally available, inexpensive parameter—as an independent predictor in the present study offers a more immediately translatable finding for resource-limited settings.

This study has several limitations that merit acknowledgment. First, the retrospective, single-center design limits the generalizability of the findings and introduces the potential for selection bias and incomplete data capture. Second, the sample size, while adequate for the primary analyses (n = 226), yielded a relatively small imaging subgroup (n = 92) with markedly unequal group sizes for the neurological dysfunction comparison (84 vs. 8), which limits the statistical power to detect differences in imaging parameters and increases the risk of type II error. Third, the diagnosis of pure SAE relied on clinical judgment by the examining neurologist, and despite systematic exclusion criteria, some degree of subjectivity in the attribution of encephalopathy etiology is inherent, particularly in patients with overlapping metabolic derangements. Fourth, more specific biomarkers of brain injury (S100β, NSE) and advanced neuroimaging modalities (MRI) were not available, which would have provided greater sensitivity in detecting subtle structural and functional brain abnormalities. Fifth, brain CT visual rating scales (GCA, Fazekas, MTA Scheltens) were assessed by a single investigator; inter-rater reliability was not evaluated, although these visual scales have generally shown acceptable inter- rater agreement in the literature. Finally, survival was assessed only at hospital discharge, and longer-term outcomes including cognitive recovery, functional status, and post-discharge mortality were not captured.

This study highlights the high burden of neurological dysfunction in emergency department sepsis patients and identifies simple, routinely available clinical, laboratory, and imaging markers associated with pure sepsis-associated encephalopathy. Prospective, multicenter validation studies are warranted to confirm these associations, develop composite bedside prediction tools, and ultimately determine whether early identification of SAE-prone patients can translate into targeted interventions that improve neurological outcomes and survival.

## Data availability

The datasets used and/or analysed during the current study are available from the corresponding author on reasonable request.

## Author contributions

FS and ES conceptualized the study, collected and curated the clinical data, performed the statistical analysis, and drafted the manuscript. SF performed the neurological examinations, established the diagnoses of neurological dysfunction and pure sepsis-associated encephalopathy, retrospectively reviewed all brain CT scans, and contributed to data interpretation. DB supervised the study design, contributed to data interpretation and critical revision of the manuscript, and provided final approval of the submitted version. All authors read, revised, and approved the final version of the manuscript and agree to be accountable for all aspects of the work.

## Conflicts of interest statement

The authors declare that they have not conflict of interest. Author Scarlatescu Ecaterina, one of the Editorial Board members of JMJ was not involved in the peer review or decision-making process for this paper.
